# Deep learning-based 3D leukocyte differentiation using label-free higher harmonic generation microscopy

**DOI:** 10.1186/s12967-026-08225-8

**Published:** 2026-05-12

**Authors:** Mengyao Zhou, Patrick José González, Tamara Dekker, Shiqi Zhang, Leonoor S. Boers, Hélène B. van den Heuvel, Annemiek Dijkhuis, Iris A. Simons, Jan Willem Duitman, Marie Louise Groot

**Affiliations:** 1 Faculty of Science, Department of Physics, Laserlab, Vrije Universiteit Amsterdam, De Boelelaan, Amsterdam, 1105, 1081HV The Netherlands; 2 Department of Experimental Immunology, Amsterdam UMC, location University of Amsterdam, Amsterdam, The Netherlands; 3 Amsterdam Infection & Immunity, Inflammatory Diseases, Amsterdam, The Netherlands; 4 Intensive Care Medicine, Amsterdam UMC, location University of Amsterdam, Amsterdam, The Netherlands; 5 Department of Pulmonary Medicine, Amsterdam UMC, Location University of Amsterdam, Meibergdreef 9, Amsterdam, The Netherlands; 6 Department of Genetics and Cell Biology, MHeNs -Research Institute for Mental Health and Neuroscience, Maastricht University Medical Centre, Maastricht, 6229 ER The Netherlands

**Keywords:** Broncho-alveolar lavage fluid (BALF), Blood fraction, Deep learning, Higher harmonic generation microscopy (HHGM), Leukocyte differentiation

## Abstract

**Background:**

Both in clinical practice and translational research, cell differentiation of leukocytes provides important diagnostic information and insights into pathophysiological mechanisms. The current gold-standard method for bronchoalveolar lavage fluid (BALF) analysis involves histochemical staining of cytospins, followed by manual morphological quantification. This approach however is labor-intensive, time-consuming, and highly operator-dependent, limiting its efficiency and throughput. This study proposes a deep learning framework for rapid, automated 3D leukocyte differentiation using label-free higher harmonic generation microscopy (HHGM).

**Methods:**

3D leukocyte imaging was performed with label-free HHGM in a few minutes. Two deep learning models, ResNet 3D-50 and Vision Transformer (ViT) 3D, were trained, validated and tested for leucocyte differentiation on both BALF and blood fraction samples from 16 interstitial lung disease (ILDs) and 19 acute respiratory distress syndrome (ARDS) patients. Deep-learning model-prediction and cytospin analysis were performed by separate investigators. The results were compared using Bland-Altman analysis.

**Results:**

The proposed framework achieved accuracies above 86% for BALF and above 96% for blood samples under five-fold cross-validation. The approach shows close agreement with gold-standard cytological analyses, with mean differences of <5% across leukocyte subpopulations.

**Conclusions:**

By integrating the label-free imaging capabilities of HHGM with deep learning, this study established a fast, accurate and high-throughput leukocyte differentiation in fresh BALF and blood samples. By significantly improving efficiency and reproducibility, this technology has the potential to transform clinical workflows and advance precision medicine.

**Supplementary Information:**

The online version contains supplementary material available at 10.1186/s12967-026-08225-8.

## Background

Determining the leukocyte composition of bronchoalveolar lavage fluid (BALF) and blood is critical for diagnosing and managing various lung diseases [1–3]. For example, in acute respiratory distress syndrome (ARDS), neutrophils are the majority cells observed in both blood and BALF, reflecting the intense inflammatory response characteristic of the disease [1]. In interstitial lung diseases (ILDs), differential cell counts in BALF are used to guide the differential diagnoses of ILD. An abnormally high number of neutrophils and/or eosinophils, often referred to as “alveolitis”, are believed to reflect a lower respiratory tract inflammation [2, 4], whereas a high lymphocyte count suggests an ILD associated with granuloma formation, such as hypersensitivity pneumonitis or sarcoidosis [4]. Moreover, in eosinophilic asthma, increased eosinophil counts are frequently observed in both blood and BALF, contributing to airway inflammation and hypersensitivity, which are key features of the condition [3, 5].

Cell differentiation using histochemical staining of cytospin followed by manual microscopic examination is currently regarded as the gold standard for leukocyte analysis due to its ability to provide detailed morphological analyses [6, 7]. However, this approach is highly labor-intensive, time-consuming, and requires well-trained personnel. As a result, outcomes may vary significantly based on the observer’s expertise and experience. Flow cytometry is an alternative way to automatically differentiate leukocytes with high accuracy, but it generally cannot provide detailed cellular information and relies on labeling with multiple antibodies against specific cell markers to achieve high classification accuracy [8–10]. Despite being accepted by experts [8, 11, 12], this technique involves substantial costs for equipment (flow cytometer) and sample preparation materials (i.e., multiple antibodies). Higher harmonic generation microscopy (HHGM), which integrates third harmonic generation (THG) and multiphoton excited autofluorescence (MPEF) microscopy, shows great potential for bridging this gap as it features 3D label-free and real-time imaging capabilities [13–15]. Applied to BALF and blood samples, this approach offers distinct advantages, such as minimal sample preparation, high throughput, and no requirement for highly trained personnel.

THG is sensitive to local variations in third-order nonlinear susceptibility, refractive index, and dispersion. Its signal is enhanced at interfaces and optical inhomogeneities that are similar in size to the beam focus [16, 17]. Due to the nonlinear dependence of signal generation on laser intensity, THG is confined to the focal region, giving it intrinsic axial optical sectioning capability. It enables near-complete visualization of tissue morphology, particularly excelling in imaging water-lipid interfaces, such as cell membranes and lipid-rich components [18]. MPEF, such as two-photon excited autofluorescence (2PEF) imaging, can detect mitochondria-related metabolic coenzymes such as NADH and FAD in various pathological conditions, including precancers [19]. Additionally, three-photon excited autofluorescence (3PEF) imaging can detect serotonin and NADH [20]. These modalities can be integrated and generate signals simultaneously, providing comprehensive subcellular information [21, 22].

For the resulting high-throughput and high-content images, equally fast and automated image analysis tools are required. There are deep learning frameworks that work well for identifying labelled cells in 2D sections [23–28]. However, 2D analyses on depth sectioned images may introduce bias as the number of detected cells can be either underestimated or overestimated depending on the depth sampling, and their size may be underestimated. Furthermore, 2D depth-sectioned images provide only partial information about the cell, potentially leading to errors in cell identification. Therefore, a 3D analysis of 3D volume measurements is required. When it comes to 3D cell analyses, two main challenges arise. First, annotating large numbers of cells is painstaking and labor-intensive. Second, the complex architecture of deep learning neural networks for big data, demands substantial computational resources to train and process images within a reasonable timeframe.

Therefore, in this study, we developed a computational pipeline in which ResNet 3D-50 and Vision transformer (ViT) 3D were trained to autonomously quantify the cell populations (neutrophils, eosinophils, lymphocytes, macrophages/monocytes) in fresh, label-free BALF and blood fraction samples without human intervention. This approach was validated and tested on BALF and blood fraction samples from ILD and ARDS patients, collected during routine clinical practice.

## Methods

### Sample collection and preparation

BALF and blood fraction samples were collected for clinical/diagnostic purposes from patients with ARDS and ILD, each following specific collection protocols, described below.

### BALF sample

In ARDS patients admitted to the ICU at both locations of the Amsterdam UMC (AMC and VUMC) who exhibited no respiratory improvement, routine diagnostic bronchoscopy with BALF was performed according to standardized protocols. The decision to perform BAL was made by joint expert opinion during multidisciplinary team meetings. During bronchoscopy, the bronchoscope was wedged into a subsegment of a lung lobe at a site identified by high-resolution computed tomography (HRCT) or chest CT imaging. Four times 20 mL 0.9% NaCl were instilled into a single lung segment, with the recovered fractions collected separately. One milliliter of the first two fractions was reserved for label-free imaging.

For ILD patients, BALF was collected by instilling eight times 20 mL 0.9% NaCl followed by gentle aspiration. One milliliter of the final fraction was allocated for label-free imaging.

**Fig. 1:**
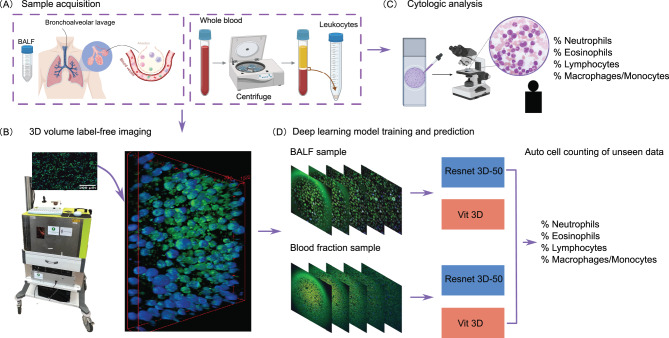
Diagrammatic illustration of overall workflow. (**A**) Sample acquisition of BALF and blood fraction sample. (**B**) 3D volume label-free imaging using HHGM. (**C**) Standard cytospin processing with DQ staining. (**D**) Training and prediction using deep learning models

Following collection (Fig. 1A), BALF samples were processed following standard cytology protocols at the diagnostic lab of Amsterdam UMC (location AMC), resulting in Diff-Quick (DQ, RAL Diagnostics) stained cytospin slides (Fig. 1C). In every sample, at least 500 cells were evaluated and classified as macrophages, lymphocytes, neutrophils, eosinophils, or other by an experienced lab technician using a standard light microscope at 40× magnification.

### Blood sample

Blood samples were only available from ARDS patients. Venous blood was collected into heparinized tubes for blood fraction analysis. Following blood collection, samples were first centrifuged to separate the plasma, which was subsequently removed. The cell pellet was treated with erylysis buffer to remove red blood cells. To this end, the cell pellet was slowly resuspended in 25 mL ice-cold erylysis buffer (ratio 1:2.5) and incubated on ice for 15 minutes. Next, cells were centrifuged (450 g, 8 °C for 10 minutes) and washed with ice-cold PBS supplemented with 0.5% BSA. This protocol typically removes around 95% of the red blood cells.

After the final wash, the leukocyte-rich fraction was resuspended and counted using a Coulter DxH500 hematology analyzer (Beckman Coulter, Brea, USA), followed by the BALF protocol (see above) to perform differential classification into monocytes, lymphocytes, neutrophils, eosinophils, and other cell types.

In parallel, 1 ml of each sample, containing approximately 0.5 million cells, were transported on ice to our lab (Imaging Center at Amsterdam UMC, location VUMC) for 3D label-free imaging, which was performed within half an hour after arrival. The sample was pipetted to a petri-dish (μ-Dish 35 mm, high, Ibidi GmbH) and placed in a portable HHGM for 3D volume label-free imaging (Fig. 1B).

In total, we collected 34 BALF samples and 19 blood fraction samples from 16 ILD patients, 1 asthma patient and 19 ARDS patients. The detailed characteristics of the patients and samples are shown in Table 1.

**Table 1:** Patient characteristics

Sample Type		BALF (N = 34)	Blood (N = 19)
Patient condition		ILD ^a^ (N = 16)	ARDS (N = 19)
		Asthma ^b^ (N = 1)	
		ARDS (N = 17)	
Age, mean (min, max)		54 (18, 81)	41 (18, 77)
Sex	Female, n (%)	14 (41.2)	8 (42)
	Male, n (%)	20 (58.8)	11 (58)
Smoking status	Never, n (%)	14 (40)	9 (47)
	Former, n (%)	2 (7.5)	1 (5)
	Current, n (%)	16 (47.5)	7 (37)
	Unknow, n (%)	2 (5)	2 (11)
^a^ILD Subtypes			
ILD (N = 16)	Unclassifiable ILD (N = 6)		
	Vasculitis (N = 2)		
	Familial pulmonary fibrosis (N = 1)		
	Fibrotic hypersensitivity pneumonitis (N = 4)		
	Cryptogenic organizing pneumonia (COP) (N = 1)		
	Idiopathic non-specific interstitial pneumonia (iNSIP) (N = 1)		
	Non-fibrotic hypersensitivity pneumonitis (N = 1)		
^b^Asthma Subtype			
Asthma (N=1)	Eosinophilic asthma (N=1)		

### HHGM setup

Figure 1 B shows the HHGM setup (co-developed with Flash Pathology B.V.) and a 3D rendering of a BALF sample. In this study, the microscope was upgraded to allow for simultaneous signal acquisition from all channels, eliminating the deviations that arose from the sequential signal collection used in the earlier setup [21, 28]. Briefly, a femtosecond-pulsed laser with a center wavelength of 1070 nm was used to scan the sample. The generated signals were separated using dichroic mirrors and filters and detected in the epi-direction. In this study, we collected THG signals at 350–360 nm, 2PEF signals at 562–665 nm, and 3PEF signals at 380–420 nm (in an initial subset of experiments (*n* = 13), a filter with broadband range of 398–501 nm was used).

3D data were collected through depth scanning at 1 ${\rm{\mu }}$m intervals, covering a total depth of 20 ${\rm{\mu }}$m, with a field of view of 400 × 400 ${\rm{\mu }}$m^2^. This imaging depth was sufficient to resolve cells settled at the bottom of the petri-dish, see Fig. 4 F. Five regions of interest were scanned per sample, with each depth scan completed within 30 seconds.

### Data processing

Twenty depth-scanned images were saved as a single TIFF file, (C, D, W, H) = (3, 20, 1000, 1000), using ImageJ (Fiji version 1.53). To remove the glass interface artifacts in THG channel, we applied rolling ball background subtraction with a radius of 50 pixels. Next, contrast enhancement was performed using contrast-limited adaptive histogram equalization (CLAHE). The data were then standardized from ${v_i}$ to $v_i'$, where ${v_i}$ denotes the pixel intensity value in channel $i$. The standardization was performed using the channel-specific mean ${v_{m,i}}$ and standard deviation ${v_{sd,i}}$ (with $i = 1,2,3$ indicating the channel index), $v_i' = \left( {{v_i} - {v_{m,i}}} \right)/{v_{sd,i}}$. This normalization ensured consistency across all images for subsequent analyses.

### Model architecture, training, and interpretation

We developed two deep learning models for automated cell counting using 3D HHGM imaging data: ResNet 3D-50 and Vision Transformer (ViT) 3D.

### Model architecture and training

**Fig. 2:**
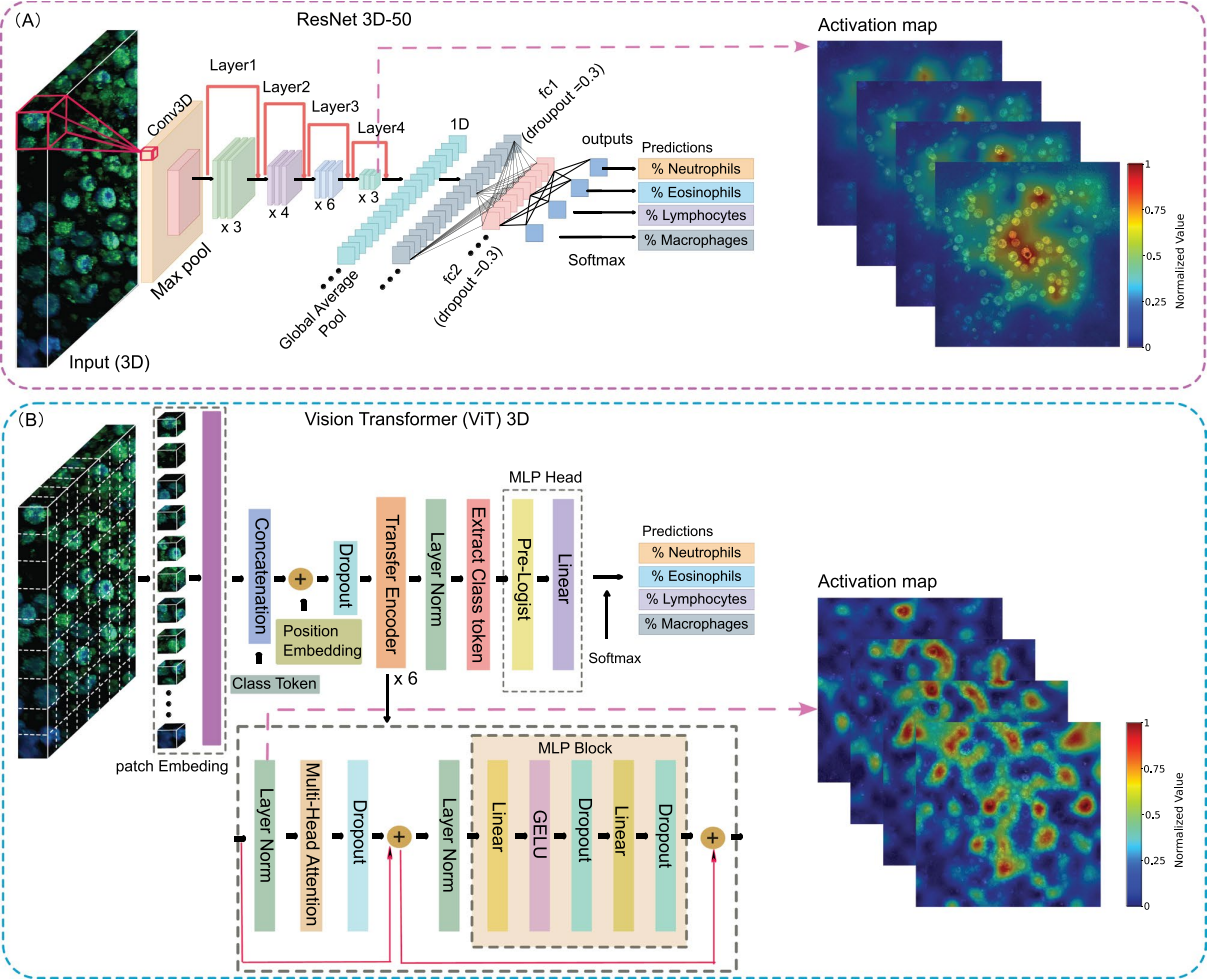
Architectural comparison of ResNet 3D-50 and ViT 3D models. The figure illustrates the key components and structural differences between the (**A**) ResNet 3D-50 and (**B**) ViT 3D architectures. ResNet 3D-50 employs 3D convolutions and residual connections to extract spatio-temporal features, while ViT 3D uses a patch-based self-attention mechanism to capture global context across the input data. Both models were utilized for 3D cell counting in this study

The first model was based on the ResNet 3D-50 architecture [29]. It consists of multiple 3D convolutional layers, pooling layers, and fully connected layers. To adapt ResNet 3D-50 for regression-based cell counting, we replaced the original classification head with a global average pooling layer followed by two fully connected layers (32 and 4 units, respectively). A SoftMax activation function was applied to predict cell counts across four discrete classes (Fig. 2A). Each fully connected layer was regularized with a dropout rate of 0.3 to prevent overfitting.

Training was performed using mean absolute error (MAE) as the loss function and optimized with stochastic gradient descent (SGD) (learning rate = 0.0012) [30]. The network was trained separately on the BALF and blood fraction datasets for 500 epochs, requiring approximately 100 hours of computation (5-fold cross-validation). The model achieving the lowest validation loss was selected for final evaluation on the hold-out test set.

In parallel, we implemented a ViT 3D model [31, 32] for comparison with ResNet 3D-50 (Fig. 2B). Input 3D images were resized to 256 × 256 × 32 × 3 and divided into non-overlapping patches of size 16 × 16 × 2 × 3, yielding 4096 patches with a patch dimension of 1536. Each patch was flattened into a one-dimensional sequence, to which class tokens and positional embeddings were added. This sequence was then processed by a transformer encoder, and the learned class token was passed through a multi-layer perceptron (MLP) head with SoftMax activation to output predictions for the four cell count classes.

The ViT 3D model was trained in a supervised manner to learn the underlying cell count distribution, using an initial learning rate of 1 × 10^− 6^. A learning rate scheduler with a step size of 1 epoch and a decay factor (γ) of 0.7 was employed to gradually reduce the learning rate. Adam optimization was used throughout training. Like ResNet 3D-50, ViT 3D was trained separately on the BALF and blood fraction datasets for 500 epochs (∼90 hours, 5-fold cross-validation), and the best-performing version on the validation set was used for test evaluation.

A five-fold cross-validation scheme was implemented to evaluate generalization performance. Each fold was tested on the same hold-out test set (20%), while the remaining data were split into training (80%) and validation (20%) sets using a fold-specific random seed (42 + fold index).

Each image was annotated with the corresponding cell percentages derived from cytological analysis. To mitigate class imbalance, we applied a label smoothing strategy [33]. To enhance model robustness and generalization, two data augmentation techniques were employed: (i) spatial flipping along the x, y, and z axes (each applied with a 50% probability), and (ii) color jittering, which randomly adjusted brightness, contrast, saturation, and hue by ±20%.

Hyperparameter optimization for both models was conducted using Optuna, minimizing validation loss across 20 trials of 20 epochs each with a median pruner strategy. The optimal hyperparameter configurations were used to train all five folds and achieve the final reported performance. Both models exhibited stable convergence of training and validation loss curves, indicating effective learning of dataset-specific patterns (see Fig. S1).

### Implementation and model interpretation

The models were implemented in PyTorch and trained on a NVIDIA A30 GPU with 25 GB RAM using the ADA HPC at the Vrije Universiteit Amsterdam. Model inference was executed on the same system and completed within approximately 5 minutes per dataset.

To interpret the learned representations and spatial attention of each network, we applied Gradient-Weighted Class Activation Mapping (Grad-CAM) [34]. Grad-CAM provides a visual explanation of model predictions by identifying image regions that contribute most strongly to the target output.

For ResNet 3D-50 model, Grad-CAM was applied to the final convolutional layer, computing the gradients of the predicted class to localize the most discriminative 3D features (Fig. 2A). For the ViT 3D model, Grad-CAM was adapted to the transformer framework by extracting gradient information from the layer normalization step of the final transformer encoder block (Fig. 2B).

### Statistical analysis

Statistical analyses were performed using Origin (Version OriginPro 2023, Academic). The results were expressed as mean ± standard deviation (SD). Bland-Altman analyses [8] were created to illustrate the average differences between the results of the cytologic analyses and models predicted results.

## Results

### Cytological analysis of leukocyte differentiation

**Fig. 3:**
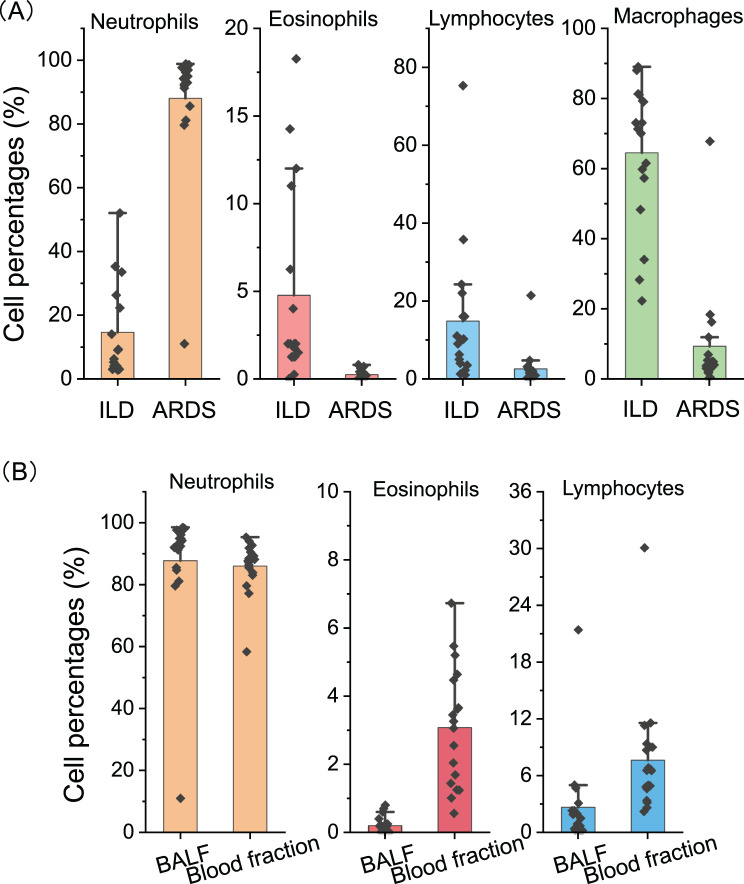
Cytological analysis of leukocyte differentiation in BALF and blood from ILD and ARDS patients. (**A**) Comparison of leukocyte composition in BALF between patients with ILD and ARDS. (**B**) Comparison of leukocyte distributions between BALF and blood in ARDS patients. Data are shown as the percentage of each leukocyte population for all cases and are presented as mean ± SD

BALF samples from ILD patients exhibited a relatively high abundance of macrophages (22% − 89%), lymphocytes (1% − 75%) and eosinophils (0% − 18%) (Fig. 3A). In contrast, ARDS BALF samples were dominated by neutrophils, which accounted for 79.6% − 98.8% (except one case 11%, BALI017) of total leukocytes (Fig. 3A).

A comparison of leukocyte subpopulations between BALF and blood fractions from ARDS patients revealed a consistently elevated proportion of neutrophils in both compartments, indicative of a systemic neutrophilic inflammatory response (Fig. 3B). Eosinophil counts were low in BALF (0–0.8%) and slightly higher in blood (0.6% − 6.7%). Lymphocyte proportions were slightly greater in blood fractions (2.2% − 30.1%) than in BALF (0.2% − 21.4%), although overall levels remained low across both sample types.

### Characterization of leukocytes via HHGM

**Fig. 4:**
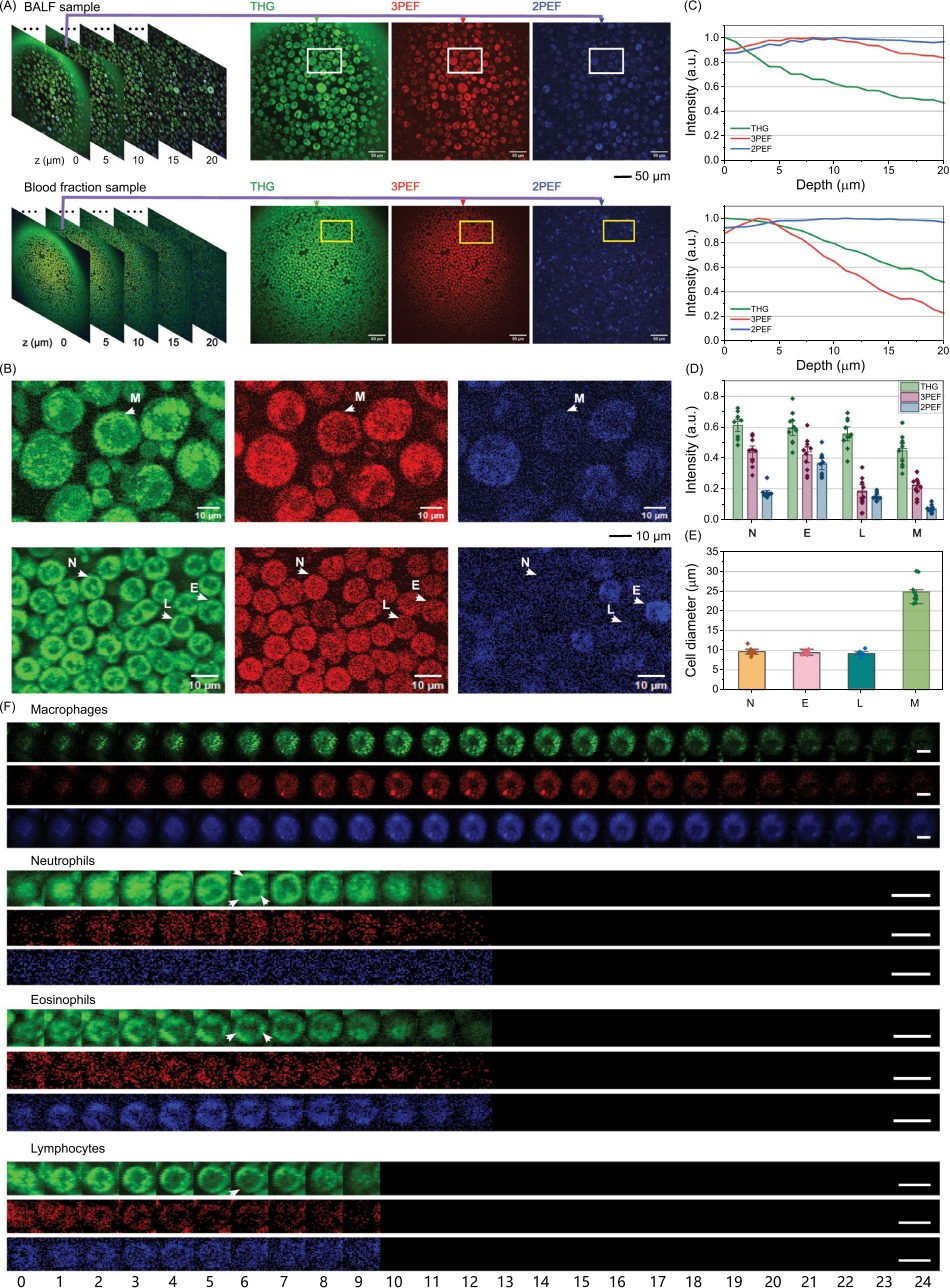
Characterization of leukocytes via HHGM. (**A**) 3D HHGM volume images displaying cellular structures in BALF and blood fraction samples acquired across 3 channels. THG signals (green) provide detailed morphological information of the cell membrane and nucleus, the 2PEF (blue) and 3PEF signals (red) originate from autofluorescence of the cytoplasm. (**B**) Zoomed-in views of the boxed areas in Fig. A, with white arrows indicating representative leukocyte types. (**C**) Depth-resolved intensity profiles of all three channels in BALF and blood samples. (**D**) Normalized THG and 2/3PEF signal intensities, and (**E**) cell diameters measured from 10 randomly selected cells per leukocyte type. (**F**) Representative 2D depth-resolved slices of individual cells. White arrows indicate distinct nuclear lobes characteristic of each cell type. Scale bar of 10 µm

Figure 4A shows representative THG and MPEF volume images of BALF and blood fraction samples acquired across three imaging channels. Corresponding representative 2D depth-resolved slices of individual cells are presented in Fig. 4F, with a scale bar of 10 µm. Randomly selected regions of interest (boxed areas) are enlarged in Fig. 4B to highlight cellular morphology. Macrophages were distinguished by their larger size, with a mean diameter of 24.8 ± 2.9 µm, whereas neutrophils, eosinophils, and lymphocytes exhibited smaller diameters ranging from 6 to 10 µm (Fig. 4E). Neutrophils typically displayed a segmented nucleus consisting of three to five connected lobes, eosinophils exhibited a bilobed nucleus, and lymphocytes and macrophages were characterized by a round, mononuclear morphology (Fig. 4B, F). Macrophages occasionally appeared multinucleated, particularly in BALF samples from patients with interstitial lung disease (ILD). In addition, both neutrophils and eosinophils showed prominent intracellular granules.

To further improve cell differentiation, spatially resolved signal information was incorporated into the analysis. Figure 4C presents the depth-dependent intensity profiles of THG, 2PEF, and 3PEF signals. THG intensity decreased with increasing imaging depth in both BALF and blood fraction samples. The 2PEF signal remained relatively stable with depth in both sample types, whereas the 3PEF signal was stable in BALF samples but decreased with depth in blood samples, likely due to increased scattering and absorption associated with the higher cellular density of blood. Among all leukocyte subtypes, eosinophils exhibited the strongest 2PEF signal, while neutrophils showed the highest 3PEF signal (Fig. 4D, F).

The distinct 2PEF and 3PEF signatures observed across leukocyte subtypes likely reflect differences in intracellular biochemistry and granule composition. Eosinophils are enriched in flavin adenine dinucleotide (FAD) within cytoplasmic granules, which exhibits strong two-photon–excited autofluorescence in the yellow–red spectral range(~500–600 nm) [35]. Accordingly, the pronounced eosinophil signal is well captured by our 2PEF detection window (562–665 nm). In contrast, neutrophils exhibit elevated levels of nicotinamide adenine dinucleotide (phosphate), NAD(P)H, associated with high metabolic activity. NAD(P)H preferentially contributes to shorter-wavelength autofluorescence and is efficiently excited under three-photon excitation(~400–500 nm) [36], resulting in dominant emission within our 3PEF detection range (380–420 nm). As illustrated in previously reported emission spectra [37], NAD(P)H emits at shorter wavelengths than FAD, further supporting the spectral separation between neutrophils and eosinophils observed in this study.

### Comparison of cytology and model predictions

**Fig. 5:**
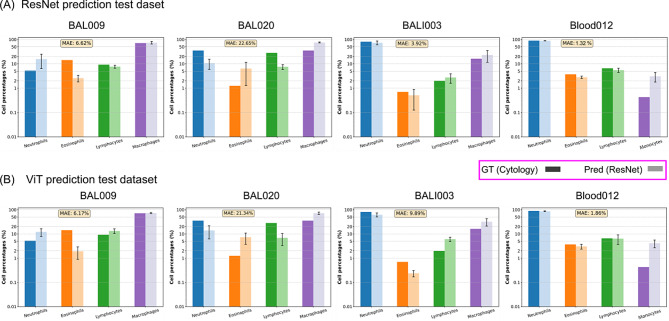
Comparison of cytology and model-predicted leukocyte distributions in representative test cases. Cytology-derived ground truth (GT) and model-predicted leukocyte percentages are shown for representative BALF (BAL009, BAL020, BAL003) and blood (Blood012) samples. Predictions from (**A**) ResNet 3D-50 and (**B**) ViT 3D models are shown. Cases span compositionally homogeneous and heterogeneous leukocyte profiles. Error bars indicate five-fold cross-validation variability; mean absolute error (MAE) is shown above each plot. BAL samples were obtained from patients with ILD (Bal)or ARDS (BALI), and blood samples from patients with acute respiratory distress syndrome

To illustrate model performance at the individual-sample level, representative test cases spanning diverse leukocyte composition profiles are shown in Fig. 5, with all test cases provided in Fig. S2. Across these examples, both ResNet and Vision Transformer (ViT) predictions closely matched cytological ground truth, capturing relative proportions of neutrophils, eosinophils, lymphocytes, and macrophages/monocytes (Fig. 5). ViT predictions showed improved agreement in samples with mixed cellular populations (BAL020), whereas ResNet performed best in compositionally homogeneous cases dominated by macrophages/monocytes or neutrophils (BAL009, BAL003, Blood012). Error bars denote variability across five-fold cross-validation, indicating robust and reproducible predictions.

When analyzed by individual leukocyte subtypes, the mean MAE across five folds for the BALF dataset using ResNet 3D-50 was 14% for neutrophils (N), 4% for eosinophils (E), 8% for lymphocytes (L), and 16% for macrophages (M) (Fig. S3 A). Corresponding MAEs for the ViT 3D model were 15% (N), 5% (E), 10% (L), and 17% (M) (Fig. S4 A). For the blood dataset, ResNet 3D-50 achieved mean MAEs of 4% (N), 2% (E), 4% (L), and 2% (monocytes) (Fig. S3C), while the ViT 3D model yielded MAEs of 5% (N), 2% (E), 4% (L), and 2% (monocytes) (Fig. S4C). All results are summarized in Table S1.

For the BALF dataset, the overall MAEs of all subtypes were consistently < 11% across all five folds for the ResNet 3D-50 model (Fig. S3B) and <12% for the ViT 3D model (Fig. S4B). Model performance was substantially higher for the blood dataset, with MAEs consistently < 3% and <4% across all folds for ResNet 3D-50 (Fig. S3D) and ViT 3D (Fig. S4D), respectively.

**Fig. 6:**
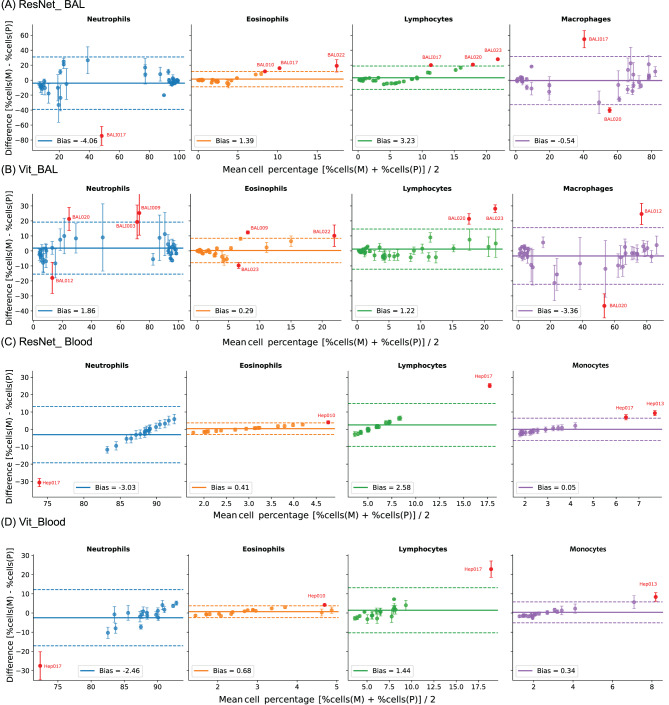
Bland–Altman analysis of agreement between cytological reference counts and model predictions. (**A**, **C**) Bland–Altman plots comparing cytological leukocyte differentials with predictions from the ResNet 3D-50 model for (**A**) 34 BALF and (**C**) 19 blood fraction samples. (**B**, **D**) corresponding Bland–Altman plots for the ViT 3D model for (**B**) 34 BALF and (**D**) 19 blood fraction samples. Solid lines denote the mean difference between cytology and model predictions, and dashed lines indicate the 95% limits of agreement. Red data points indicate outliers. Error bars represent standard deviation across folds

Agreement between cytological reference counts and model predictions was further assessed using a Bland–Altman analysis. The mean differences between cytology and ResNet 3D-50 predictions were < 5% for all leukocyte subpopulations in both BALF and blood samples (Fig. 6A, C), while the corresponding differences for the ViT 3D model were slightly smaller, at <4% (Fig. 6B, D). Subpopulation-specific differences are summarized in Table 2.

In addition, the 95% confidence intervals (CIs) of the differences between cytology and ResNet 3D-50 predictions were contained within a ± 35% margin for all leukocyte subpopulations (Fig. 6A, C). In contrast, the 95% CIs for the ViT 3D model were consistently narrower and remained within a ± 20% margin (Fig. 6B, D), indicating tighter agreement with cytological measurements and improved predictive precision. A summary of the 95% CIs for all leukocyte subpopulations is provided in Table 3.

To further elucidate the observed performance differences between architectures, class-specific activation maps were analyzed and are presented in the Supplementary Information (Fig. S5). Overall, with closely matched model sizes (4.3 M and 4.6 M parameters, see Table S1 in supplementary information), both architectures are highly parameter‑efficient and suited for resource‑constrained deployment.

**Table 2:** Summary of differences between cytological analyses and model predictions across leukocyte subpopulations

Mean Differences		Neutrophils	Eosinophils	Lymphocytes	Macrophages	Monocytes
BAL	Cytology – ResNet 3D-50	-4.1%	1.4%	-3.2%	-0.5%	-
	Cytology – ViT3D	-2.0%	-0.3%	1.2%	-3.4%	-
Blood	Cytology – ResNet 3D-50	−3.0%	0.4%	2.6%	-	0.1%
	Cytology – ViT3D	−2.5%	0.7%	1.4%	-	0.3%

**Table 3:** Summary of the lower and upper agreement limit of subpopulations between cytologic analyses and model predictions

Lower and upper agreement limit		Neutrophils	Eosinophils	Lymphocytes	Macrophages	Monocytes
BAL	Cytology & ResNet 3D-50	-40%, 35%	-14%, 16%	-18%, 20%	-32%, 32%	-
	Cytology & ViT3D	-16%, 20%	-9%, 9%	-12%, 10%	-21%, 11%	-
Blood	Cytology & ResNet 3D-50	−19%, 12%	−2%, 2%	−15%, 10%	-	−8%, 8%
	Cytology & ViT3D	−18%, 12%	−2%, 2%	−10%, 12%	-	−6%, 6%

## Discussion

Differential cell counting in body fluids such as blood and BALF is routinely used in both research and clinical practice, with BALF cytology playing a particularly important role in the diagnosis and management of ILDs. In this study, we present a label-free HHGM imaging and deep learning–based analysis pipeline for leukocyte differential quantification.

The proposed framework enables accurate quantification of leukocyte composition in both BALF and blood fraction samples, achieving accuracies above 86% for BALF and above 96% for blood samples under five-fold cross-validation. By incorporating volumetric information from label-free HHGM imaging, the approach shows close agreement with gold-standard cytological analyses, with mean differences of <5% across leukocyte subpopulations. In comparison with prior 2D HHGM studies [28], which reported accuracies exceeding 90% based on a single train–test split, the present work incorporates 3D spatial information and a more rigorous cross-validation strategy, providing a more reliable assessment of model generalizability. Although the mean cross-validated accuracy for BALF is modestly lower, multiple folds achieved accuracies above 90%, indicating performance comparable to earlier 2D approaches when evaluated under similar conditions. The comparable performance of convolutional (ResNet) and transformer-based (ViT) 3D architectures further suggests that robust leukocyte differential prediction can be achieved without explicit 3D cell segmentation, supporting the scalability and translational potential of the proposed method.

Beyond predictive performance, the proposed HHGM-based approach offers several practical advantages over conventional cytological workflows. The method operates without labeling or staining, significantly reducing sample preparation time and procedural complexity. Once configured, each sample can be scanned and analyzed within approximately 10–15 minutes, enabling near–real-time assessment in acute clinical settings such as intensive care units or bronchoscopy suites. In addition, the system requires only a small sample volume (1 mL), making it particularly well suited for situations in which specimen availability is limited. Together, these features position the proposed framework as a promising diagnostic tool, especially in resource-limited or point-of-care settings where access to conventional cytology may be restricted.

Despite these promising results, several limitations should be acknowledged. The relatively limited sample size and class imbalance may affect model performance for underrepresented leukocyte populations. To mitigate these effects, we employed data augmentation, cross-validation, and careful model optimization. Nevertheless, broader validation is required. Future work will address this limitation through multi-center data collection designed to enrich rare leukocyte populations and increase cohort diversity. In parallel, we will investigate synthetic data augmentation strategies, including generative adversarial network (GAN)-based approaches, to enhance minority class representation while preserving biologically meaningful features.

Additionally, while validation in independent ILD and ARDS cohorts supports the method’s utility in these settings, its applicability to other biological fluids—such as bronchial washes, sputum, or cerebrospinal fluid—remains to be determined. These sample types present distinct challenges, including variable cellular composition, mucus content, debris, and low cell density, which may necessitate protocol adaptations to ensure reliable performance.

We approached multi-class cell counting as a regression task rather than a classification task. While classification-based approaches are commonly employed [38–40], regression allows the model to predict continuous cell percentages, more closely reflecting the quantitative nature of leukocyte differentials. This formulation explicitly captures the relative distances between counts (e.g., 25 vs. 26 cells being closer than 25 vs. 53) [27], which is critical for accurate clinical interpretation. However, the regression framework does not explicitly incorporate spatial localization, potentially introducing false positives. Future work will explore the integration of density estimation strategies, such as convolutional neural network–based spatial heatmaps or attention-guided density maps [26], to jointly model cell counts and spatial distributions. Such approaches could provide both quantitative and qualitative insights into leukocyte organization within 3D volumes while preserving computational efficiency.

Finally, a limitation of the HHGM-based approach relative to flow cytometry, which is often part of the clinical BAL analysis, is the inability to directly assess cell surface marker expression, restricting its use in settings requiring precise immunophenotyping, such as hematologic malignancies or immune deficiencies. Marker-based techniques therefore remain indispensable for these applications. However, in routine clinical evaluation of interstitial lung disease (ILD) and acute respiratory distress syndrome (ARDS), immunophenotypic marker information is rarely used for decision-making. In ILD, the CD4/CD8 ratio is among the few markers occasionally considered, whereas biomarker-based stratification is not routinely applied in ARDS. Instead, leukocyte differential ratios constitute the primary clinically actionable metric in both contexts. Within this framework, the label-free nature of the proposed method, together with minimal infrastructure requirements and rapid analysis, offers a pragmatic and clinically aligned alternative to marker-dependent techniques.

Pre-analytical processing is another determinant of performance. Incomplete erythrocyte lysis can increase debris and residual anucleate cells, complicate segmentation, and thereby bias monocyte estimates. Future work will sensitivity analyses to benchmark monocyte estimation across a range of pre-analytical conditions and explore integration of HHGM with targeted molecular or immunophenotypic readouts to combine label-free imaging with marker-level specificity.

## Conclusions

The combination of HHGM and deep learning presents a promising approach for differentiating leukocytes in fresh, label-free BALF and blood fraction samples. This technique has the potential to accelerate the diagnostic process while reducing costs, expertise needed and overall workload .

## Electronic supplementary material

Below is the link to the electronic supplementary material.

Supplementary Material 1

## Data Availability

The datasets used during this study are available from the corresponding author on reasonable request.
