# Supplementary material for: Deep learning-based 3D leukocyte differentiation using label-free higher harmonic generation microscopy
Source: J Transl Med. 2026 May 12;24:854. doi: 10.1186/s12967-026-08225-8 (PMC13334751; doi:10.1186/s12967-026-08225-8)
Supplement: Supplementary file 1 — Supplementary Material 1 [file 12967_2026_8225_MOESM1_ESM.docx]

**Supplemental Material for “Deep Learning-Based 3D Leukocyte Differentiation Using Label-Free Higher Harmonic Generation Microscopy”**

Mengyao Zhou^1, *,†^, Patrick José González^1^, Tamara Dekker^2,3^, Shiqi Zhang^4^, Leonoor S. Boers^4^, Hélène B. van den Heuvel^4^, Annemiek Dijkhuis^2,3,5^, Iris A. Simons^2,3,5^, Jan Willem Duitman^2,3,5^, Marie Louise Groot^1^.

1. Faculty of Science, Department of Physics, Laserlab, Vrije Universiteit Amsterdam, De Boelelaan 1105, 1081HV, Amsterdam, The Netherlands. 2. Department of Experimental Immunology, Amsterdam UMC, location University of Amsterdam, Amsterdam, The Netherlands. 3. Amsterdam Infection & Immunity, Inflammatory Diseases, Amsterdam, The Netherlands. 4. Intensive Care Medicine, Amsterdam UMC, location University of Amsterdam, Amsterdam, The Netherlands. 5. Department of Pulmonary Medicine, Amsterdam UMC, location University of Amsterdam, Meibergdreef 9, Amsterdam, The Netherlands. **^†^ Present address:** Department of Genetics and Cell Biology, MHeNs -Research Institute for Mental Health and Neuroscience, Maastricht University Medical Centre, Universiteitssingel 50, 6229 ER, Maastricht, the Netherlands

***Correspondence** : [mengyao.zhou@maastrichtuniversity.nl](mailto:mengyao.zhou@maastrichtuniversity.nl)

**Supplementary Figures**

**Fig.S1. Training and validation loss curves for ResNet 3D-50 and ViT 3D models on the BALF and blood fraction datasets**. The curves show the mean across the five folds, with the shaded band representing one standard deviation. Training loss is weighted under label smoothing to mitigate data imbalance. For BALF, ResNet 3D-50 converges steadily and the validation loss stabilizes ~0.10 MAE, while ViT 3D converges more slowly but reaches ~0.08 MAE. For the blood fraction dataset, both models converge within ~50 epochs, with training and validation losses stabilizing at ~0.05 MAE.

**Fig.S2.** Comparison of cytology and model-predicted leukocyte distributions in all test datasets. Panels: (A) ResNet predictions and (B) ViT predictions. BAL samples are from ILD patients, BALI samples from ARDS patients, and blood samples are from ARDS patients. N, E, L, M denote neutrophils, eosinophils, lymphocytes, and macrophages/monocytes, respectively.

**Figure S3. Performance of the ResNet 3D-50 model for leukocyte differential prediction.** (A) MAE across five-fold cross-validation for individual leukocyte subtypes in BALF samples. (B) Overall MAE across all leukocyte subtypes for each cross-validation fold in the BALF dataset. (C) Mean MAE for individual leukocyte subtypes in blood fraction samples. (D) Overall MAE across all leukocyte subtypes for each cross-validation fold in the blood dataset. Error bars represent standard deviation across test cases.

**Figure S4. Performance of the ViT 3D model for leukocyte differential prediction.** (A) MAE across five-fold cross-validation for individual leukocyte subtypes in BALF samples. (B) Overall MAE across all leukocyte subtypes for each cross-validation fold in the BALF dataset. (C) Mean MAE for individual leukocyte subtypes in blood fraction samples. (D) Overall MAE across all leukocyte subtypes for each cross-validation fold in the blood dataset. Error bars represent standard deviation across cases.

**Figure S5 | Model activation maps for cellular composition estimation.** Representative samples were selected to span low, medium, and high proportions of neutrophils or macrophages/monocytes across BALF and blood samples For each case, class-specific activation maps are shown for ResNet (top row) and ViT (bottom row) models across leukocytes subtypes. Numeric values indicate predicted cell proportions. ResNet exhibits broad, diffuse activations, particularly in macrophage-dominant samples, suggesting reliance on coarse spatial density and texture cues. In contrast, ViT displays sparse, localized attention patterns aligned with individual cell-sized structures and distributed across the full field of view (magenta dashed circles in activation map), consistent with global contextual reasoning and implicit instance counting. These differences highlight the superior suitability of transformer architectures for estimating relative cellular composition in heterogeneous samples.

**Supplementary Table**

**Table S1. Models’ performance vs number of parameters**

| **Model** | | **Parameters (M)** | **Mean MAE (across 5-fold cross-validation)** | | | | |
| --- | --- | --- | --- | --- | --- | --- | --- |
|  |  |  | Neutrophils | Eosinophils | Lymphocytes | Macrophages | Monocytes |
| ResNet 3D-50 | BAL | 4.3  4.3 | 14% | 4% | 8% | 16% | - |
|  | Blood |  | 4% | 2% | 4% | - | 2% |
| ViT3D | BAL | 4.6  4.6 | 15% | 5% | 10% | 17% | - |
|  | Blood |  | 5% | 2% | 4% | - | 2% |
